# Nanoarchitectonics for Wide Bandgap Semiconductor Nanowires: Toward the Next Generation of Nanoelectromechanical Systems for Environmental Monitoring

**DOI:** 10.1002/advs.202001294

**Published:** 2020-09-24

**Authors:** Tuan‐Anh Pham, Afzaal Qamar, Toan Dinh, Mostafa Kamal Masud, Mina Rais‐Zadeh, Debbie G. Senesky, Yusuke Yamauchi, Nam‐Trung Nguyen, Hoang‐Phuong Phan

**Affiliations:** ^1^ Queensland Micro and Nanotechnology Centre Griffith University Nathan QLD 4111 Australia; ^2^ Electrical Engineering Department University of Michigan Ann Arbor MI 48109 USA; ^3^ Department of Mechanical Engineering University of Southern Queensland Springfield QLD 4300 Australia; ^4^ Australian Institute of Bioengineering and Nanotechnology The University of Queensland St Lucia QLD 4072 Australia; ^5^ NASA JPL California Institute of Technology Pasadena CA 91109 USA; ^6^ Department of Aeronautics and Astronautics Stanford University Stanford CA 94305 USA

**Keywords:** environmental monitoring, nanoarchitectonics, nanofabrication, nanosensors, semiconductor nanowires

## Abstract

Semiconductor nanowires are widely considered as the building blocks that revolutionized many areas of nanosciences and nanotechnologies. The unique features in nanowires, including high electron transport, excellent mechanical robustness, large surface area, and capability to engineer their intrinsic properties, enable new classes of nanoelectromechanical systems (NEMS). Wide bandgap (WBG) semiconductors in the form of nanowires are a hot spot of research owing to the tremendous possibilities in NEMS, particularly for environmental monitoring and energy harvesting. This article presents a comprehensive overview of the recent progress on the growth, properties and applications of silicon carbide (SiC), group III‐nitrides, and diamond nanowires as the materials of choice for NEMS. It begins with a snapshot on material developments and fabrication technologies, covering both bottom‐up and top‐down approaches. A discussion on the mechanical, electrical, optical, and thermal properties is provided detailing the fundamental physics of WBG nanowires along with their potential for NEMS. A series of sensing and electronic devices particularly for environmental monitoring is reviewed, which further extend the capability in industrial applications. The article concludes with the merits and shortcomings of environmental monitoring applications based on these classes of nanowires, providing a roadmap for future development in this fast‐emerging research field.

## Graphical abstract

Wide bandgap semiconductor nanowires with unique properties and advanced fabrication/integration technologies set a new class of nanosensing and electronic applications. Implementation of these smart devices such as gas sensors, photodetectors, wearable devices, strain sensors, and energy harvesters into environmental monitoring systems offers promising solutions for critical environmental challenges.

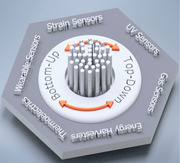

## 1 Introduction

Rapid industrialization and negative impacts from human activities over the past decades have raised serious environmental concerns, such as increased water and air pollution, extreme temperature swings, and global warming. Therefore, protecting and improving our environment have become more urgent today than ever before.^[^ ^1^, ^2^ ^]^ In this regard, considerable research efforts have been devoted to developing advanced materials and technologies, which aim to attain appropriate solutions for critical environmental challenges.^[^ ^3^, ^4^ ^]^ Among them, monitoring the environmental conditions and inspecting industrial processes using sensors and optoelectronics is an innovative method that can help in reducing harmful impacts and preventing catastrophic failures.^[^ ^5^, ^6^, ^7^, ^8^, ^9^, ^10^, ^11^ ^]^ A key merit of this approach is the advancements in nanofabrication technologies along with the emergence of new classes of materials that have enabled the mass production of sensing devices, which are of low‐cost but yet posing enhanced sensitivity and reliability. These devices are suitable for deployment in Internet of Things (IoTs) and more specifically in Internet of Sensors.^[^ ^12^, ^13^, ^14^, ^15^, ^16^, ^17^, ^18^ ^]^

To date, the Internet of Sensors for environmental monitoring mainly relies on standardized inorganic semiconductor materials, especially silicon (Si) due to its well‐established physics, matured fabrication processes, and worldwide availability.^[^ ^19^, ^20^, ^21^, ^22^, ^23^ ^]^ In this context, thin‐film‐based sensors have been the preferred choice over bulk materials due to their cost‐effectiveness and compatibility with standard micro/nanomachining technologies.^[^ ^24^, ^25^ ^]^ However, despite these huge advantages, many existing sensors based on thin‐film semiconductors still exhibit significant deficiencies such as poor sensitivity and unstable operation due to the limitation of their surface‐to‐volume ratios.^[^ ^26^, ^27^ ^]^ To improve and optimize their overall performance, new scaling‐down methods have been developed, employing both top‐down and bottom‐up approaches to create a wide array of 1D structures. Among the various forms of 1D nanostructures, such as nanotubes, nanofibers, nanorods, and nanoribbons, semiconductor nanowires have been emerging as ideal candidates for the next generation of sensors and optoelectronic devices, particularly for environmental inspection as well as for scavenging wasted energies.^[^ ^28^, ^29^, ^30^, ^31^, ^32^, ^33^, ^34^ ^]^ Nanowires are defined as normal electrical wires having a ratio of length to width larger than 1000 nm.^[^ ^35^ ^]^ At the nanoscale level, nanowires exhibit numerous advantages compared their bulk counterparts, including tunable properties, large surface‐to‐volume ratios, excellent sensitivity, low power consumption, and the ability to integrate different functional elements into a single chip.^[^ ^36^, ^37^, ^38^, ^39^ ^]^ For example, piezoresistance of top‐down and bottom‐up fabricated Si nanowires was found to be much more significant compared to that of bulk Si.^[^ ^40^, ^41^ ^]^ Moreover, the quantum confinement of nanowires may result in exotic properties in sensing and optoelectronic devices such as tunable bandgap and fast operation speed.^[^ ^42^, ^43^, ^44^ ^]^ Another vital benefit of nanowires is that, unlike conventional thin‐film structures, lattice mismatch strain in nanowires can be relieved at the free surfaces by elastic deformation without dislocation defects.^[^ ^45^, ^46^, ^47^ ^]^ These advantages of nanowires offer exciting opportunities for their integrations on diverse substrates for the development of high‐performance sensing platforms for environmental monitoring.

**Figure 1:**
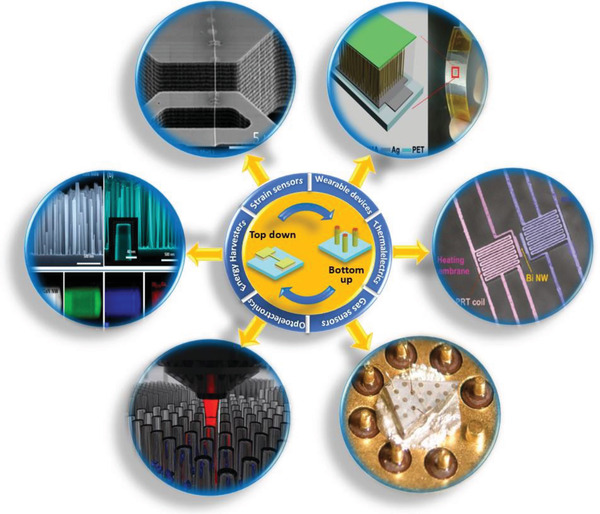
Overview of wide bandgap nanowires‐based sensing and optoelectronic devices for diverse applications in environmental monitoring: strain sensors. Reproduced with permission.^[^ ^293^ ^]^Copyright 2019, IOP Publishing. Wearable devices. Reproduced with permission.^[^ ^294^ ^]^ Copyright 2015, Wiley‐VCH. Thermosensors. Reproduced with permission.^[^ ^295^ ^]^ Copyright 2011, Springer Nature. Optoelectronics. Reproduced with permission.^[^ ^296^ ^]^ Copyright 2017, American Chemical Society. Gas sensors. Reproduced with permission.^[^ ^297^ ^]^ Copyright 2006, MDPI Publishing. Energy harvesters. Reproduced with permission.^[^ ^298^ ^]^ Copyright 2018, MDPI Publishing.

Wide bandgap (WBG) materials, widely known as the third‐generation semiconductors, have been a hot spot of modern electronic devices. The utilization of WBG nanowires, such as those made of SiC, GaN, and diamond, has attracted increasing attention owing to their superior chemical inertness and mechanical properties together with interesting electrical behaviors.^[^ ^48^, ^49^, ^50^, ^51^, ^52^, ^53^ ^]^ In particular, the chemical inertness of WBG semiconductors offers the reliable and long‐term operation, while their large energy gap allows for high‐temperature operation as well as unique functionalities (e.g., UV detection) that cannot be achieved with conventional Si nanowires (**Figure** **1**). A considerable number of review papers are available on the incredibly rapid progress of such semiconductor nanowires and their diverse applications.^[^ ^54^, ^55^, ^56^, ^57^ ^]^ However, very few of them have considered the current status of WBG nanowires‐based sensing devices with an emphasis placed on environmental monitoring applications. On the basis of these observations, this article provides a comprehensive review of the research activities focusing on the use of WBG semiconductor nanowires of SiC, group III‐nitrides, and diamond for environmental monitoring applications. First, the state‐of‐the‐art developments and remaining shortcomings of various fabrication techniques and growth methods using both bottom‐up and top‐down approaches are introduced. Second, recent efforts on determining and improving mechanical, optical, electrical, and thermal properties of SiC, group III‐nitrides, and diamond nanowires associated with sensing and optoelectronic devices are highlighted. Thereafter, a series of the most important applications in environmental monitoring, such as in sensors, photonics platforms, and energy‐harvesting devices based on these nanowires is delineated. Finally, the review paper concludes with an outlook on future opportunities and solutions to overcome the remaining challenges in the field of WBG semiconductor nanowire‐based devices for environmental monitoring.

## 2 Material Development and Nanomachining Technologies

Semiconductor nanofabrication technologies are generally classified into the two well‐known methods: bottom‐up and top‐down. The bottom‐up approach is an additive process in which atoms and molecules are the basic building blocks to construct the sophisticated nanostructures. The top‐down approach, on the other hand, is a subtractive process, where excess material is physically or chemically removed to produce the desired objects with a controllable shape and size.^[^ ^58^ ^]^ Each of these approaches has its own advantages and limitations for the fabrication of semiconductor nanowires.^[^ ^59^ ^]^ For example, a benefit of the bottom‐up method is the flexibility to synthesize a variety of nanowires with tailored properties. However, the most significant drawbacks of this approach are the limitation of large‐scale uniformity and integration into functional devices. Unlike the bottom‐up approach where the grown nanowires might be distributed randomly, the top‐down approach enables precise positioning to produce highly aligned nanowire arrays across the wafer and thus offers the large‐scale‐integration for high‐performance nanoelectronic circuits with standard industry processes. Nevertheless, the resolution limitation and high operation cost are the main disadvantages of the top‐down technology. Therefore, either one of these approaches or the combination of both approaches will be employed for the fabrication of nanowires‐based electronic devices depending on particular applications.

### 2.1 Synthesis and Growth of Bottom‐Up Nanowires

**Figure 2:**
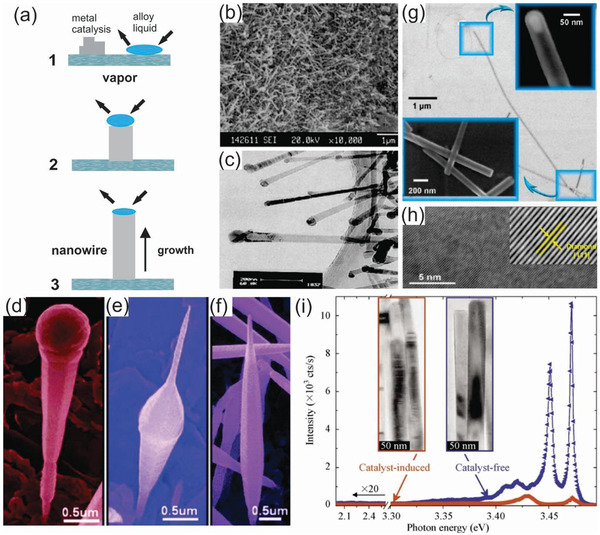
a) Schematic illustration of vapor–liquid–solid (VLS) nanowire growth mechanism including three stages 1 (alloying), 2 (nucleation), and 3 (axial growth). Reproduced with permission.^[^ ^70^ ^]^ Copyright 2001, American Chemical Society. b,c) SEM and TEM images of SiC nanowires growth by VLS method, respectively ((a) to (c) reproduced with permission.^[^ ^76^ ^]^ Copyright 2002, Elsevier. d–f) SEM images of the SiC nanowires showing different structures ranging from an Eiffel‐tower, spindle, and modulated shape, respectively, depending on the growth conditions. Reproduced with permission.^[^ ^78^ ^]^ Copyright 2008, American Chemical Society. g) Electron microscopy of diamond nanowires encased within a carbon nanotube shell. The inset in (d) shows a catalyst embedded inside the tip of the nanowire. h) The high‐resolution TEM image of a single diamond nanowire. Inset in (h) shows a zoomed‐in view of the crystalline structure of cubic diamond (111) surface. (d) and (e) reproduced with permission.^[^ ^77^ ^]^ Copyright 2010, American Chemical Society. i) Low‐temperature PL spectra of GaN nanowires grown by catalyst‐induced and catalyst‐free growth methods. Insets in (i) show the corresponding TEM images showing the stacking faults for the case of catalyst‐induced growth method. Reproduced with permission.^[^ ^92^ ^]^ Copyright 2010, Tsinghua University Press.

Various effective methods have been developed for the bottom‐up growth of semiconductor nanowires.^[^ ^60^, ^61^, ^62^, ^63^, ^64^ ^]^ Among them, the vapor–liquid–solid (VLS) mechanism has been the most widely exploited technique since first introduced by Wagner and Ellis for large whisker growth in 1960s.^[^ ^65^, ^66^, ^67^, ^68^, ^69^ ^]^ In principle, the VLS mechanism relies on a three stage‐process, including alloying, nucleation, and axial growth stage (**Figure** **2**a).^[^ ^70^ ^]^ In the alloying stage, a catalytic liquid alloy phase is formed on the substrate upon annealing. Subsequently, this alloy phase can rapidly absorb a vapor of precursors to its supersaturation level in the nucleation stage. Finally, nanowire growth can proceed from nucleated seeds at the solid/liquid interface in the growth stage. The VLS method has successfully produced SiC, gallium nitride (GaN), and diamond nanowires.^[^ ^71^, ^72^, ^73^, ^74^, ^75^ ^]^ For instance, SiC nanowires with 20 nm diameter and 20 µm length were grown at elevated temperature using aluminum as a catalyst (Figure 2b,c).^[^ ^76^ ^]^ Notably, one of the key merits of the bottom‐up nanowire growth based on the VLS mechanism is that various types of nanowires can be synthesized in a controllable manner depending on the proper choice of the substrates, catalysts, and growth parameters (e.g., pressure, temperature, and time). As such, Wang et al. demonstrated the possibility to synthesize SiC nanowires with an Eiffel‐tower, spindle, and modulated shape by varying the pressure of the precursors in the VLS growth process, which are completely different from cone or tip‐shaped SiC nanowires commonly reported by other research groups as shown in Figure 2d–f.^[^ ^78^ ^]^ Men et al. reported that SiC nanowires synthesized by VLS method were long and thin under nickel (Ni)‐based catalysis but they were short and thick with Fe‐based catalysis.^[^ ^79^ ^]^ Krishnan et al. reported that the growth direction of SiC nanowires was dictated by the crystallographic orientation of the 4H‐SiC substrates, which could be exploited to enable growth of highly aligned SiC nanowires.^[^ ^81^ ^]^ Low et al. investigated the morphological and structural characteristics of GaN nanowires synthesized by Ni‐catalyzed chemical vapor deposition (CVD) method with respect to the growth temperatures. The results revealed that straight and smooth GaN nanowires with the best elemental composition were achieved at the optimal growth temperature of 950 °C.^[^ ^80^ ^]^ On the other hand, diamond nanowires were grown on a Si substrate using the CVD process based on the VLS mechanism with an iron (Fe) catalyst. The role of Fe catalyst in this approach is to enhance diamond nucleation as previously demonstrated by several studies.^[^ ^82^, ^83^ ^]^ These diamond nanowires are straight, thin, and long over tens of micrometers with a high crystallinity and structural uniformity (Figure 2g,h).^[^ ^77^ ^]^ Another main advantage of the bottom‐up method based on the VLS process is in situ doping by incorporating desired dopant precursors in the synthesis procedure. This is very beneficial to the fabrication of high‐performance optical and electronic devices because such the bottom‐up growth method eliminates the requirement for additional destructive steps (e.g., ion implantation) to introduce additional charge carriers to the as‐grown nanowires.^[^ ^64^, ^84^ ^]^ For instance, Gao et al. reported, for the first time, the synthesis of Al‐doped SiC nanowires. The doping levels of these nanowires were controlled by tailoring the Al concentrations in the precursors, which caused redshifts of the photoluminescence bands.^[^ ^85^ ^]^ In another example, Liu et al. synthesized high‐quality GaN nanowires doped with Si, which showed a distinct blueshift from the bulk bandgap emission.^[^ ^86^ ^]^

Although having these great benefits, the bottom‐up nanowire synthesis based on the VLS process has some inherent drawbacks, which are basically induced by the presence of foreign metallic elements serving as catalysts in the VLS process. These metallic particles may result in the formation of unavoidable defects and thus lead to negative effects on optical and electronic properties of the as‐grown nanowires, which has been proven by several research groups.^[^ ^87^, ^88^, ^89^, ^90^, ^91^ ^]^ For example, Karakostas et al. compared GaN nanowires grown by either catalyst‐free or catalyst‐induced methods utilizing Ni seeds and found that the catalyst‐induced nanowires contain many more basal‐plane stacking faults and their photoluminescence is much weaker in comparison to that of the nanowires synthesized by the catalyst‐free route (Figure 2i).^[^ ^92^ ^]^ A more critical problem is the incorporation of metallic particles within nanowires that typically causes detrimental impacts on device performance. For example, although gold (Au) is the most widely employed catalyst in the VLS process, it exhibits a fast diffusion from the surface into the underlying Si substrate and then acts as a deep‐level trap, reducing the carrier lifetime, which is inherently incompatible with semiconductor technology.^[^ ^93^, ^94^ ^]^ To overcome these daunting challenges, a considerable number of studies have been devoted to establishing new strategies for the catalyst‐free synthesis of wide bandgap semiconductor nanowires. These catalyst‐free routes are based on both vapor–liquid–solid and vapor–solid mechanisms employing a wide range of synthesis techniques, such as CVD,^[^ ^95^, ^96^, ^97^, ^98^, ^99^ ^]^ molecular beam epitaxy (MBE),^[^ ^100^, ^101^, ^102^, ^103^, ^104^, ^105^ ^]^ arc discharge processes,^[^ ^106^, ^107^ ^]^ carbothermal reduction,^[^ ^108^, ^109^ ^]^ thermal evaporation,^[^ ^110^, ^111^ ^]^ and oxide‐assisted methods.^[^ ^62^, ^112^ ^]^ Among them, CVD and MBE‐based growth methods are considered as the most frequently used techniques to produce high‐quality crystalline nanowires of WBG semiconductor materials, especially SiC, diamond, and group III‐nitride. For example, Tchernycheva et al. reported on the catalyst‐free growth of GaN nanowires by a plasma‐assisted MBE process. The GaN nanowires synthesized by this work are free of crystalline defects and thus, exhibit excellent optical properties.^[^ ^113^ ^]^

### 2.2 Nanomachining Process for Top‐Down Nanowires

**Figure 3:**
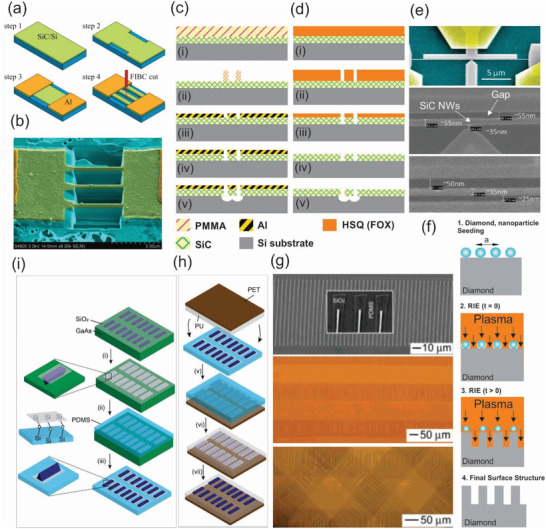
a,b) Schematic illustration of the fabrication of SiC NWs using focused ion beam and SEM image of the as‐fabricated SiC NWs in which the thickness, width, and length of the NWs were 300 nm, 300 nm, and 5 µm, respectively. Reproduced with permission.^[^ ^121^ ^]^ Copyright 2016, IEEE Publising. c–e) Illustrations of the lift‐off and negative‐mask surface nanomachining processes for making the SiC NWs using EBL and A 20 µm long SiC NW with a 15 µm wide lateral gate and a point‐contact gate, respectively ((c)–(e) reproduced with permission.^[^ ^127^ ^]^ Copyright 2010, American Chemical Society. f) Schematic plots of fabrication of diamond nanowires using a combination of EBL and reactive ion etching technique with nanodiamond particles as a hard mask. Reproduced with permission.^[^ ^130^ ^]^ Copyright 2008, American Chemical Society. g) SEM images of GaAs wire arrays bonded to PDMS via the SiO_2_ stripes (top) and optical images of GaAs wire arrays on PU/PET sheets with different numbers of layers of wires: single layer (middle) and triple layers (bottom). h,i) Schematic illustration of the process of generating and transfer printing GaAs wire arrays onto plastic substrates. (g)–(i) reproduced with permission.^[^ ^134^ ^]^ Copyright 2004, American Chemical Society.

Among various popular top‐down nanofabrication techniques in semiconductor technology, focused ion beam (FIB) is one of the most powerful tools for surface modifications and prototyping functional structures of semiconductor materials at the micro‐ and nanoscale.^[^ ^114^, ^115^ ^]^ The principle of FIB is almost identical to scanning electron microscopy but uses a beam of ions in place of electrons. The basic components of a standard FIB system include the ion column, the work chamber, the vacuum system, and the workstation for the user interface. FIB applies highly focused ion beams to modify the target surface via the sputtering process. By properly controlling the energy and intensity of the ion beams, FIB offers a great opportunity to perform very precise nanomachining for producing sophisticated nanostructures.^[^ ^116^, ^117^ ^]^ On the basis of these advantages, FIB has been widely employed to fabricate WBG semiconductor nanowires out of prepatterned microstructures for the development of sensing and optoelectronic devices.^[^ ^118^, ^119^, ^120^ ^]^ For example, Phan et al. investigated the piezoresistive effect of 3C‐SiC nanowires fabricated using the FIB technique for mechanical sensing applications.^[^ ^121^ ^]^ This finding demonstrated a new pathway to mass production of SiC nanowires on Si wafers and their integrations with CMOS devices. Detailed information of the resulting SiC nanowires and processing steps for the top‐down fabrication of SiC nanowires using FIB are shown in **Figure** **3**a,b.

Along with the FIB, electron beam lithography (EBL) is also a well‐known technique for designing and creating semiconductor nanowires, which are impossible with traditional photolithography methods due to the diffraction limits of light.^[^ ^122^, ^123^, ^124^ ^]^ A typical EBL system consists of a column of electron optics for producing and tuning the beam, control electronics, and a sample stage. The EBL technique allows the creation of nanoscale patterns by directly modifying the solubility of a resist material that is sensitive to electron beams. The resolution of EBL depends on the spot size of the focused beam and the scattering of primary and secondary electrons in the resist film and the substrate. The main advantage of this technique is the ultrahigh resolution of sub‐10 nm that can be achieved via the well‐controlled process.^[^ ^125^ ^]^ EBL has been used as a standalone tool, or in combination with other top‐down techniques for manufacturing of WBG semiconductor nanowires integrated into sensing devices. In one of the early studies, Juhasz et al. reported a precisely controlled fabrication of SiC nanowires with their widths approaching the 10 nm regime using a combined process of EBL and electrochemical size reduction.^[^ ^126^ ^]^ More interestingly, Roukes et al. developed very thin SiC nanowires using two different top‐down nanofabrication processes based on EBL for low voltage nanoelectromechanical switches, as shown in Figure 3c,d.^[^ ^127^ ^]^ These processes enable the thinnest nanowires as well as air gaps in their devices with thickness and widths of 16–25 nm and a minimum gap of 9 nm, as shown in Figure 3e. The extremely tiny air gap and widths allowed for an excellent contact‐mode in nanomechanical switches, offering very low turn‐on voltages down to the level of ≈1 V along with exceptionally short switching times in the sub‐microsecond range.

The combination of different well‐known lithography methods has created and established several efficient protocols that advance the top‐down fabrication of WBG semiconductor nanowires.^[^ ^128^, ^129^, ^130^, ^131^ ^]^ Examples of this approach includes the work of Yang and colleagues, which formed vertically aligned diamond nanowires with precisely controllable geometrical properties using nanodiamond particles as a hard mask for subsequent anisotropic reactive ion etching.^[^ ^130^ ^]^ This etching mask in the form of diamond nanoparticles was deposited on a single crystalline diamond film and then, reactive ion etching was applied to remove unprotected areas, as shown in Figure 3f. As a result, vertically aligned diamond nanowires with optimized geometrical dimensions as small as 10 nm in diameter and a median distance of 11 nm were achieved. Using a similar combination of EBL and dry etching, Liao et al. successfully fabricated suspended single‐crystal diamond nanowires for high‐performance nanoelectromechanical switches that can operate under harsh environments.^[^ ^131^ ^]^

Recent advancements in printing technology and flexible electronics have also triggered the development of WBG semiconductor nanowires integrated onto a soft platform. Flexible WBG nanowire electronics offer many interesting properties for future multifunctional sensing devices, such as biocompatibility, light weight, transparency, and flexibility. Unfortunately, the direct growth of desired nanowires on plastic substrates is almost impossible due to the intolerance of the soft substrate to the elevated temperatures required for the growth process. The limitation of the bottom‐up approach for flexible nanowire electronics has propelled studies on scalable top‐down methods for transferring semiconductor nanowires onto plastic substrates at a high degree of structural perfection for their usage in biocompatible applications, such as in implantable and wearable sensing devices.^[^ ^132^, ^133^, ^134^, ^135^, ^136^ ^]^ Among these methods, dry‐transfer methods represent an effective top‐down pathway to print a variety of highly ordered arrays of semiconductor nanowires onto plastic substrates. In a pioneering study, the Rogers group developed a dry‐transfer printing process employing a PDMS‐stamp to drop top‐down fabricated GaAs nanowire arrays onto plastic substrates with good crystallographic orientation (Figure 3g).^[^ ^127^ ^]^ The whole process is schematically shown in Figure 3i,h and involves several key steps including: i) anisotropic chemical etching of a GaAs wafer using SiO_2_ strips as mask producing GaAs nanowire arrays; ii) contact between PDMS and SiO_2_ leading to the formation of siloxane bonds via a condensation reaction; iii) peeling the PDMS stamps from the GaAs substrates lifts off the wires; iv) polishing the remaining GaAs wafer which enables a new cycle of wire fabrication and lift‐off; v) contact between a PDMS stamps (with GaAs wires) and liquid film of PU cast on the PET sheet which causes the PU to flow and conforms to the wires; vi) curing PU with UV light which bonds the wires to the PU and the PU to the PET, and then peeling off the PDMS stamp which leaves the wires embedded in PU; vii) buffered oxide etching which removes the SiO_2_ mask.^[^ ^132^ ^]^

### 2.3 Hybrid Fabrication Techniques

**Figure 4:**
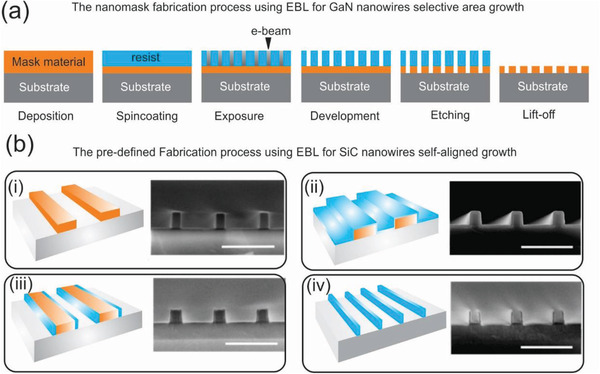
a) Illustration of the nanomask fabrication process using EBL for GaN nanowires selective area growth. Reproduced with permission.^[^ ^138^ ^]^ Copyright 2015, Amercian Chemical Society. b) Illustration of the predefined substrate fabrication process using EBL for SiC nanowires self‐aligned growth and corresponding SEM images for each processing step. Reproduced with permission.^[^ ^141^ ^]^ Copyright 2018, MDPI Publishing.

Considering the significant advantages and limitations in each top‐down and bottom‐up method, hybrid fabrication technologies that leverage the strength of the two approaches have been extensively investigated to create extremely low dimensional nanowires with precisely controllable properties. Hybrid fabrication processes offer an unprecedented possibility in moving semiconductor nanowires from the laboratory to the factory for large‐scale fabrication of practical sensing devices. In this context, the bottom‐up growth methods based on CVD and MBE techniques are widely employed in combination with conventional lithography and etching techniques to achieve precise control over the growth process of various WBG semiconductor nanowires.^[^ ^137^, ^138^, ^139^, ^140^, ^141^ ^]^ As an interesting example, the Stutzmann group fabricated a nanomask on a diamond by EBL substrate prior to the growth of GaN nanowires by using MBE.^[^ ^138^ ^]^ This mask allows the growth of perfectly homogeneous GaN nanowire arrays with miniaturized diameters and distances. **Figure** **4**a schematically shows the processing steps for the fabrication of the nanomask. Another excellent example is the work of Tabassum et al., which synthesized ultrahigh self‐aligned SiC nanowires with tailored properties at predetermined locations on Si wafers without the requirement for a lithographic pattern transfer technique. This synthesis route used EBL to form ribbon arrays of hydrogen silsesquioxane as predefined patterns on Si substrates. The SiC nanowires were subsequently grown on such patterns using a well‐controlled thermal CVD process and ion etching techniques (Figure 4b).

## 3 Properties of Wide Bandgap Semiconductor Nanowires

In comparison to their bulk counterparts, nanowires are expected to exhibit distinct properties induced by their large ratio of surface‐to‐volume and quantum confinement effects due to their small diameters. These unique properties of nanowires play a vital role in the operation mode of novel multifunctional sensing devices. Therefore, this section provides an overview on the electrical, mechanical, optical, and thermal properties of WBG semiconductor nanowires that can be utilized for niche sensing applications. **Table** **1** represents important properties of several WBG semiconductor materials commonly employed for the fabrication of sensing devices for environmental monitoring applications.

**Table 1:** Important properties of several WBG semiconductor materials for fabricating sensing devices^[^ ^304^, ^305^, ^306^ ^]^

Materials	GaAs	GaN	6H‐SiC	4H‐SiC	3C‐SiC	Diamond
Bandgap, *E*_g_ [eV]	1.43	3.45	3.03	3.26	2.35	5.45
Dielectric constant, *ε*_r_	13.1	9	9.66	10.1	9.6	5.5
Electric breakdown field, *E*_c_ [kV cm^−1^]	400	2000	2500	2200	1500	10 000
Electron mobility, *μ_n_* [cm^2^ V^−1^ s^−1^]	8500	1250	500	1000	900	2200
Hole mobility, *μ*_p_ [cm^2^ V^−1^ s^−1^]	400	850	101	115	40	850
Thermal conductivity, *λ* [W cm^−1^ K^−1^]	0.46	1.3	4.9	4.9	4.9	22
Saturated electron velocity, *ν*_sat_ [×10^7^ cm s^−1^]	1	2.2	2	2	2	2.7

## 4 Electrical Properties

**Figure 5:**
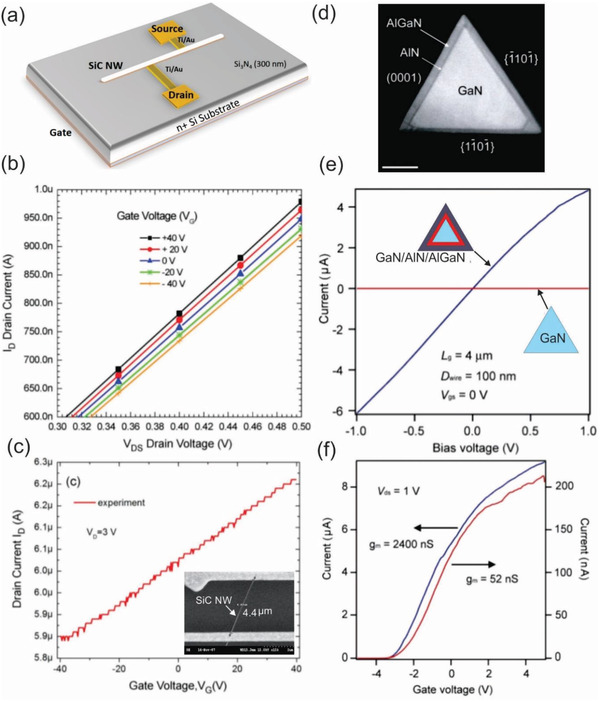
a) Schematic view of an FET device and experimental characteristics of the single SiC nanowire‐based FET devices. b) *I*_D_–*V*_DS_ curves, showing n‐type semiconductor behavior and c) *I*_D_–*V*_G_ curve at *V*_DS_ of 3 V. (a)–(c) reproduced with permission.^[^ ^143^ ^]^ Copyright 2008, IEEE Publishing. Inset in (c) shows an SEM image of SiC nanowires‐based FET. d) STEM image of a GaN/AlN/AlGaN nanowire cross‐section. e) *I*_DS_ –*V*_DS_ characteristics recorded on 100 nm diameter GaN/AlN/Al_0.25_Ga_0.75_N (blue) and GaN (red) nanowires. Insets in (e): schematics of the GaN and GaN/AlN/AlGaN nanowires. f) *I*_DS_–*V*_GS_ transfer characteristics of the same GaN/AlN/Al_0.25_ Ga_0.75_ N (blue) and GaN (red) nanowires for *V*_DS_ = 1 V. (d) and (e) reproduced with permission.^[^ ^169^ ^]^ Copyright 2006, Amercian Chemical Society.

The electrical properties of semiconductor nanowires are generally characterized by several main figures‐of‐merits. For nanowire‐based field‐effect transistors (FETs), the figures‐of‐merits include transconductance, mobility, subthreshold slope, and the ratio between on and off currents (*I*_on–off_), which can be derived from a detailed measurement and analysis of the device. In general, a higher value of these figures‐of‐merits indicates a better electrical performance of nanowires‐based FETs. Compared to the most common CMOS material, Si, understanding of the physics of WBG semiconductor nanowires such as SiC, GaN, and diamond is still immature, and thus there is plenty of room for further exploration and improvement via the measurements of FETs.^[^ ^142^, ^143^, ^144^, ^145^, ^146^, ^147^, ^148^, ^149^, ^150^, ^151^ ^]^ Notably, most of these studies show the intrinsic n‐type semiconductor behavior of SiC nanowires, which is attributed to the high density of donor states under the conduction band edge resulting from structural defects like stacking faults. As an example, Rogdakis et al. analyzed a SiC nanowire‐based FET device and found that the device with the Ohmic contact could not be switched off even at high gate bias voltages up to −40 V. This weak gating effect indicates high carrier concentration induced by unintentional doping close to the metallic limit (**Figure** **5**a–c).^[^ ^143^ ^]^ These results are responsible for the poor device performance of SiC nanowire‐based FETs. To overcome these shortcomings, the improved electrical properties of SiC nanowires have been achieved by surface modifications^[^ ^152^, ^153^, ^154^ ^]^ or proper controlling doping concentrations and doping elements.^[^ ^85^, ^146^ ^]^

In the case of GaN nanowires, various research studies have reported that unintentionally doped GaN nanowires were n‐type resulting from the intrinsic nitrogen vacancies, which were observed using the *I*–*V* characteristics of a top‐gate GaN nanowires‐based FETs.^[^ ^155^, ^156^, ^157^ ^]^ Similar to SiC nanowires, several strategies have been developed to enhance electrical properties of GaN nanowires for high‐performance electronic devices, such as doping,^[^ ^158^, ^159^, ^160^, ^161^ ^]^ surface functionalization,^[^ ^162^, ^163^ ^]^ and constructing heterostructure nanowires.^[^ ^164^, ^165^, ^166^ ^]^ The combination of different dissimilar group III‐nitride semiconductor nanowires is currently viewed as a powerful pathway for the fabrication of heterostructure nanowires‐based devices bearing superior properties, which were impossible to achieve previously.^[^ ^167^, ^168^ ^]^ A pioneering study reported by the Lieber group constructed dopant‐free GaN/AlN/AlGaN radial nanowire heterostructures for the implementation of high electron mobility transistors.^[^ ^169^ ^]^ This work also compared the electrical properties of FETs fabricated from the GaN/AlN/Al_0.25_Ga_0.75_N (Figure 5d) and undoped GaN nanowires. The *I*_DS_–*V*_DS_ and *I*_DS_ *–V*_GS_ characteristics demonstrate the heterostructure‐based FET device behaves as a n‐type depletion and confirm the accumulation of electron carriers. On the other hand, undoped GaN nanowire‐based FETs exhibit 50‐time smaller transconductance value and high resistivity at *V*_GS_ = 0, compared to that of the GaN/AlN/A_0 25_Ga_0 75_N heterostructures (Figure 5e,f). Because both heterostructures and GaN nanowires are undoped, the enhanced electrical properties of the heterostructure‐based FET could be attributed to the formation of a confined free electron gas in the radical nanowire heterostructure through a band structure engineering.

## 5 Mechanical Properties

**Figure 6:**
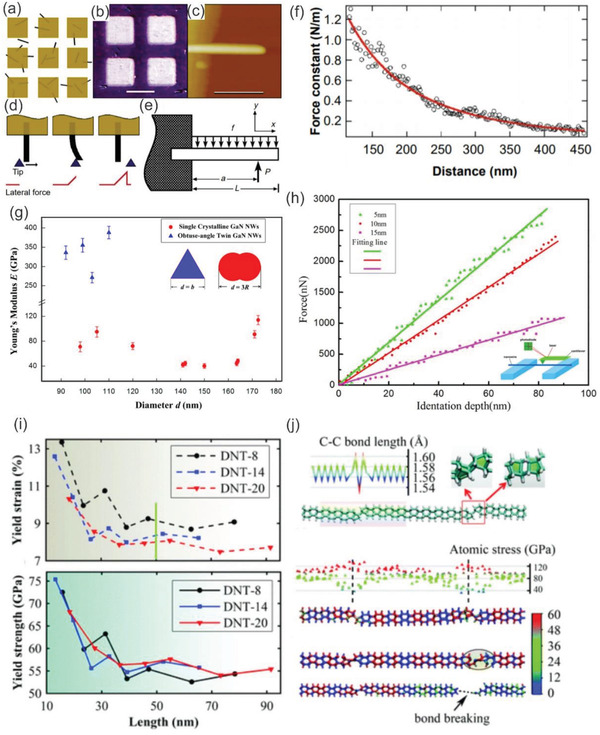
Illustration of the method employed to characterize mechanical properties of SiC nanorods. a) SiC nanorods were pinned by deposition of a grid of square SiO pads. b) Optical image of a sample showing SiO pads and MoS_2_ substrate. c) An AFM image of a 35.3 nm diameter SiC nanorod. The scale bar is 500 nm. d) Schematic of beam bending with an AFM tip. e) Schematic of a pinned beam with a free end. f) Curve fitting of the measured force constant *k*(*x*) on position *x* along the axis of the same nanorod. (a)–(f) reproduced with permission.^[^ ^172^ ^]^ Copyright 2017, Wiley‐VCH. g) Young's modulus *E* with diameter *d* of GaN nanowires from resonance experiments. Reproduced with permission.^[^ ^178^ ^]^ Copyright 2015, American Chemical Society. h) Force–displacement curves and Young's modulus of SiO_2_‐coated SiC nanowires with different SiO_2_ thickness. Reproduced with permission.^[^ ^179^ ^]^ Copyright 2016, Nature Publishing Group. i) Estimated yield strain (top) and yield strength (bottom) of the diamond nanothread (DNT) constructed by different units. j) The C—C bonding length distribution in the DNT along with the pentagons and hexagons at the defect (top), the virial atomic stress distribution along the length direction which clearly shows the stress concentration at the defect region (middle) and the bond breaking configuration at the pentagon, which indicates the failure of the DNT from the defect (bottom). (i) and (j) reproduced with permission.^[^ ^188^ ^]^ Copyright 2016, Royal Society of Chemistry.

An in‐depth understanding of the mechanical properties of semiconductor nanowires is essential for the design, fabrication, and application of nanowire‐based devices, as the nanowires are expected to behave differently from their bulk counterparts with their dimensions shrunk to nanoscale. Single‐crystalline nanowires are expected to be considerably stronger than their larger dimensional counterparts, which is attributed to a reduction in the number of defects per unit length, as defects usually result in mechanical failure.^[^ ^170^, ^171^ ^]^ The mechanical properties of nanowires are basically evaluated via several key parameters, such as elasticity, strength, and toughness. Taking advantages of nanofabrication techniques, several approaches have been developed to characterize the mechanical properties of various semiconductor nanowires.^[^ ^172^ ^]^ Wong et al. utilized atomic force microscopy (AFM), for the first time, to determine the mechanical properties of individual, structurally isolated SiC nanorods that were pinned at one end on a solid surface.^[^ ^172^ ^]^ The bending force was monitored as a function of displacement along the unpinned lengths. According to their results, the 30 nm diameter SiC nanorod had a Young's modulus of 610–660 GPa, which matched well with the value of ≈600 GPa theoretically predicted for a single SiC crystal along the 〈111〉 direction. **Figure** **6**a–f illustrates detailed information of this approach and the experimental data for calculating the Young's modulus. Other approaches have also been employed to characterize the mechanical properties of nanowires, such as field emission microscopy,^[^ ^173^ ^]^ in situ transmission electron microscopy (TEM)^[^ ^174^, ^175^ ^]^ and SEM.^[^ ^176^ ^]^ Huang et al. measured the deformation and the fracture strength of individual GaN nanowires in real time using a TEM combined with scanning probe microscope. The authors pointed out that, although local plasticity was observed frequently, global plasticity was not present, indicating the overall brittle nature of GaN nanowires. Dislocations are generated at a point with stress value near the fracture strength of the nanowire, ranging from 0.21 to 1.76 GPa.^[^ ^177^ ^]^

Controlling and tuning the mechanical properties of nanowires is imperative for the fabrication of nanowire‐based devices. For this goal, it is highly vital to determine and understand parameters, which may strongly influence the mechanical properties of nanowires during material growth and nanomachining processes. A large number of influencing parameters has been addressed, including, but not limited to defects, size, temperature, doping levels, growth orientations, and surface coating.^[^ ^178^, ^179^, ^180^, ^181^, ^182^ ^]^ Dai et al. investigated elastic properties of GaN nanowires with different structures, employing an in situ electron microscopy. The experimental results implied that for a single crystal structure aligned in the longitudinal 〈120〉 direction, the measured Young's modulus varies from 271 to 388 GPa in the diameter range of 92 to 110 nm. These data are consistent with the reported theoretical and experimental values of bulk GaN in the same direction. On the other hand, the Young's modulus value of the obtuse‐angle twin structures was measured from 40 to 114 GPa, as shown in Figure 6g. This result revealed that the elastic modulus of GaN nanowires is dependent on the relative orientations and the volume fractions of the planar defects.^[^ ^178^ ^]^ In another work, Ma et al. demonstrated that the oxide layer has a certain effect on the modulus of SiC nanowires, despite the thickness of the sheath being very thin. The authors suggested that the thickness of the oxide coating layer changes by ≈5 nm, the Young's modulus produces 16% change on average, as shown in Figure 6h.^[^ ^179^ ^]^

From a practical point of view, it is important not only to characterize the mechanical properties of nanowires, but also to examine their dynamic responses, such as elastic‐plastic response^[^ ^183^, ^184^, ^185^ ^]^ and brittle–ductile transition.^[^ ^186^, ^187^, ^188^ ^]^ An insightful understanding of mechanisms of such mechanical responses is highly demanded to design nanowire‐based devices bearing tunable mechanical properties. Recently, Zhan et al. employed large‐scale molecular dynamics simulations to demonstrate that the sp^3^ bonded diamond nanothread can transition from brittle to ductile behavior by varying the length of the polybenzene sections (Figure 6i,j). This transition arises from the hardening process of the Stone–Wales transformation defects under tension. The Stone–Wales defect acts like a grain boundary that interrupts the consistency of the polybenzene rings in the diamond nanothread structure.^[^ ^188^ ^]^ The dynamic transition in nanowires could open new opportunities to manipulate their mechanical properties at the nanoscale level, which is not achievable with the bulk platform.

### 5.1 Optical Properties

**Figure 7:**
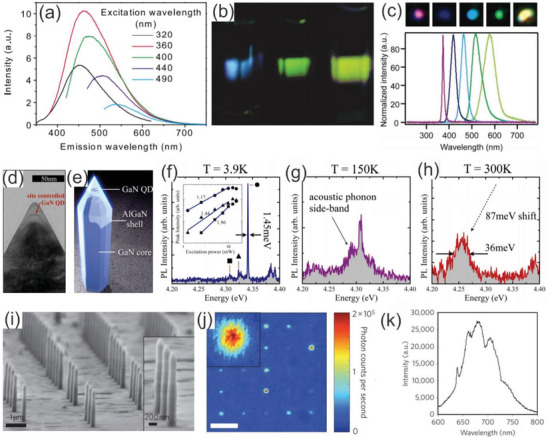
a) PL spectra of 3C‐SiC nanocrystallites taken under five different excitation wavelengths ranging from 320 to 490 nm. b) Emission photos from the 3C‐SiC samples under excitation by three different wavelengths taken by a digital camera. (a) and (b) reproduced with permission.^[^ ^191^ ^]^ Copyright 2005, American Physical Society. c) Normalized EL spectra of GaN‐based core/multishell nanowire radical heterostructures recorded from five forward‐biased multicolor nanowire LEDs (bottom) and the optical microscopy images collected from around p‐contact of LEDs in forward bias (up). (c) reproduced with permission.^[^ ^198^ ^]^ Copyright 2005, Amercian Chemical Society. d,e) TEM clearly showing the formation of a single quantum dot near the tip of a single nanowire and the corresponding illustration, respectively. f–h) Emission spectra from a single nanowire‐quantum dot measured at 3.9, 150, and 300 K measured with excitation powers of 5, 10, and 15 mW, respectively. (d)–(h) reproduced with permission.^[^ ^199^ ^]^ Copyright 2014, Amercian Chemical Society. i) An SEM image showing an array of the typical ≈200 nm diameter diamond nanowires used in the experiment. j) Confocal microscope image of a square array of nanowire devices (scale bar, 5 µm k) PL spectrum shows the nitrogen vacancy center zero‐phonon line at ≈637 nm and phonon sideband from 640 to 780 nm. (i)–(k) reproduced with permission.^[^ ^200^ ^]^ Copyright 2010, Nature Publishing Group.

The optical properties of wide bandgap semiconductor nanowires have been a subject of intensive research because the nature of a wide bandgap material offers tremendous advantages for the development of optoelectronic devices. In particular, quantum‐confinement effects are expected to generate various remarkable optical features once the diameter of nanowires is shrunk below a critical value (the Bohr radius), which do not exist in their bulk counterparts. In the early work of the field of optoelectronic using WBG nanowires, Liang et al. performed photoluminescence (PL) measurement of SiC nanowires and found two broad PL emission peaks at the center wavelength of 340 and 440 nm.^[^ ^189^ ^]^ The authors claimed that the blueshift in the emission peak could be attributed to the quantum confinement effect in comparison to that of the previous studies of PL in bulk SiC.^[^ ^190^ ^]^ However, the mechanism of the quantum confinement effect was still unclear and not fully addressed in this report. Following this work, Wu et al. demonstrated, for the first time, the quantum confinement effect of 3C‐SiC nanocrystallites with size ranging from 1 to 6 nm suspended in aqueous solution. The room‐temperature photoluminescence under excitation with different wavelengths showed the emission band maximum ranging from 440 to 560 nm, as shown in **Figure** **7**a.^[^ ^191^ ^]^ Notably, the emission intensities were so strong that the emission spots were clearly visible by naked eyes (Figure 7b). Similar to the electrical and mechanical properties, there are several primary factors which influence the optical properties of WBG semiconductor nanowires, such as morphologies, microstructures, size, defects, strain, pressure, doping, growth orientations.^[^ ^192^, ^193^, ^194^, ^195^, ^196^, ^197^, ^198^ ^]^ For example, Wu et al. reported that the PL spectra strongly depend on the variation in morphologies of SiC nanowires, while the blueshift with respect to bulk SiC is attributed to the microstructures and defects within the nanowires.^[^ ^192^ ^]^ Chin et al. observed crystal‐orientation dependent PL in GaN nanowires. The authors claimed that the surface states can trap photoexcited carriers, leading to the blue‐shifted PL of *a*‐axis GaN nanowires compared to other orientations.^[^ ^193^ ^]^

The understanding of the factors that manipulates the optical properties of nanowires has prompted the development of efficient and reliable approaches, such as defect engineering, surface modifications, heterostructures, to design and fabricate a new class of novel photonic devices with tunable optical properties.^[^ ^198^, ^199^, ^200^, ^201^, ^202^, ^203^ ^]^ For example, Qian et al. synthesized single crystal n‐GaN/In*_x_*Ga_1‐_ *_x_*N/GaN/p‐AlGaN/p‐GaN nanowire heterostructures with a well‐defined radical modulation of doping, composition, and thickness. In this work, electroluminescence measurements demonstrated that under the forward bias the core/multishell nanowires function as light‐emitting diodes, with tunable emission from 365 to 600 nm (Figure 7c) and high quantum efficiencies.^[^ ^198^ ^]^ The Arakawa group invented single photon emitters for on‐chip quantum communication by combining site‐controlled quantum dots of GaN and GaN/AlGaN core–shell nanowires grown by selective area CVD. The GaN quantum dots were successfully embedded in the core–shell nanowires by a short growth step, demonstrated by a high‐resolution TEM image and illustrated by a cartoon (Figure 7d,e)). Figure 7f–h presents the µ‐PL spectrum measured from a single device operating at 3.9, 150, and 300 K, respectively. These results prove that the use of a WBG nanowire, such as GaN/AlGaN, enables exciton confinement at high temperature. On the other hand, the implementation of small and site‐controlled quantum dots eliminates spectral contamination caused by emission overlap from neighboring quantum dots and prevents significant spectral contamination at high temperatures from states related to the same quantum dots.^[^ ^199^ ^]^ Another pioneering work reported by the Loncar group focused on the fabrication of a single‐photon source composed of a nitrogen‐vacancy center in a diamond nanowire. This device produces ten times greater flux than bulk diamond devices, while reducing the consuming power by tenfold. EBL and reactive‐ion etching were employed to realize ≈200 nm diameter and ≈2 µm long diamond nanowires with straight, smooth sidewalls, as shown in Figure 7i. The nitrogen‐vacancy centers were randomly created during the growth process and then isolated mechanically through the etching process. Figure 7j presents conformal microscope image of a square array of nanowire devices. Evidently, single‐photon sources with the best performance appear red due to their high photon count rates, while light blue and yellow spots correspond to nanowires with no embedded nitrogen‐vacancy centers and nanowires containing a weakly coupled nitrogen‐vacancy center, respectively. PL spectrum of photons collected from a typical diamond nanowire (Figure 7k) indicates the photostability of the nitrogen‐vacancy center embedded in the diamond nanowires.^[^ ^200^ ^]^ These exciting results are expected to open‐up a new class of photonic devices based on nanostructured diamond, essential for the next generation of nanoelectromechanical systems (NEMS) and sensing systems.

### 5.2 Thermal Properties

**Figure 8:**
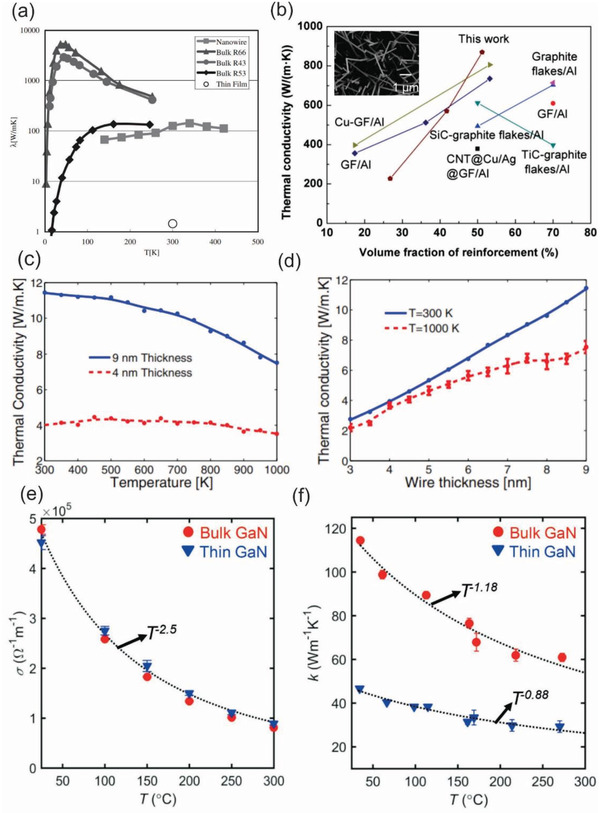
a) Comparison of thermal conductivities of SiC nanowires with their bulk and film counterparts. Reproduced with permission.^[^ ^207^ ^]^ Copyright 2008, Old City Publising. b) Comparison of thermal conductivity along the *xy* direction of the SiC nanowire–graphite reinforced Al laminar composites and other materials reinforced Al composites. Reproduced with permission.^[^ ^210^ ^]^ Copyright 2019, Royal Society of Chemistry. The inset in (b) show SEM images of SiC nanowires deposited on graphite surface. c,d) Thermal conductivity of 4 and 9 nm thick GaN nanowires as a function of temperature and thermal conductivity as a function of the wire thickness at 300 and 1000 K, respectively. (c) and (d) reproduced with permission.^[^ ^211^ ^]^ Copyright 2014, American Physical Society. e,f) Temperature‐dependent electrical conductivity and temperature‐dependent thermal conductivity measured from the bulk and thin GaN samples, respectively. (e) and (f) reproduced with permission.^[^ ^216^ ^]^ Copyright 2018, Wiley‐VCH.

Thermal properties in WBG nanowires have attracted a great deal of interest from the scientific community due to its vital role in environmental applications, particularly for energy conversion and thermoelectric devices. Notably, both experimental and theoretical studies have demonstrated that the thermal conductivity of SiC nanowires can be dramatically reduced in comparison to that of their bulk counterparts due to the size confinement effect.^[^ ^204^, ^205^, ^206^, ^207^, ^208^ ^]^ When the dimension of nanowires is smaller than a critical value, the thermal conductivity will be reduced due to boundary scattering, which limits phonon mean free paths. Takahashi et al. reported on the thermal conductivity of SiC nanowires in a wide range of temperature and compared to those of the thin film and bulk counterparts. The authors found that the thermal conductivity of SiC nanowires is much greater than that of the SiC thin film by 1.44 W m^−1^ K^−1^ but smaller than pure bulk samples (**Figure** **8**a). This result can be interpreted by the formula of phonon thermal conductivity; *λ = Cυℓ*/3, where *C* is specific heat, *υ* is phonon velocity, and *ℓ* is phonon mean free path.^[^ ^207^ ^]^ In another study, Lee et al. developed the four‐point‐probe third harmonic method to measure the thermal conductivity of individual *β*‐SiC nanowires and found that the thermal conductivity of the nanowire was about 86.5 ± 3.5 W m^−1^ K^−1^, which is approximately four times lower than that of bulk *β*‐SiC materials (350 W m^−1^ K^−1^).^[^ ^209^ ^]^ Considering the fact that lower thermal conductivity combined with a good electrical conductivity results in an enhancement in thermoelectric effect, SiC nanowires can be utilized for energy harvesting at elevated temperatures. Furthermore, mixing WBG nanowires with composites is considered as an effective tool to enhance the thermal properties of reinforced materials. Chang et al. demonstrated that the use of SiC nanowires deposited on graphite film surface to reinforce Al‐based laminar composites results in a better thermal conductivity compared to other carbon materials reinforced Al‐based composites (Figure 8b).^[^ ^210^ ^]^

Parameters that significantly affect the thermal conductivity of WBG nanowires have also been extensively explored. Lee et al. reported that the thermal conductivity of SiC nanowires is dependent on their structures. The team measured the thermal conductivity of single and double SiC nanowires having the same diameter and found that the thermal conductivities were 82 ± 6 and 73 ± 5 W m^−1^ K^−1^ for the single and double SiC nanowires, respectively.^[^ ^119^ ^]^ In another work, Papanikolaou theoretically investigated the dependence of the thermal conductivity on the cross‐section of SiC nanowires and the influence of different wire surfaces on the thermal transport using nonequilibrium classical molecular dynamics simulation. A larger cross‐section of the nanowires shows a better thermal conductivity. Furthermore, Si terminated SiC nanowires exhibit a lower thermal conductivity than C terminated one due to stronger scattering on a more disordered Si surface compared to that of C surface.^[^ ^208^ ^]^ Davoody et al. reported a comprehensive computational study of the thermal properties of GaN nanowires with a wide range of thickness values (3 to 9 nm) and at various temperatures (300 to 1000 K). Nanowires with a larger diameter possess a higher thermal conductivity, but also exhibit more profound decrease in the conductivity at elevated temperatures (Figure 8c). While, the thermal conductivity of GaN nanowires as a function of the wire thickness reveals that the thermal conductivity increases with increasing wire thickness at both room and high temperatures (Figure 8d).^[^ ^211^ ^]^ Moreover, Wang et al. reported that the thermal conductivity of GaN nanowires also exhibits a strong orientational dependence by applying nonequilibrium atomistic simulation methods using Stillinger–Weber potentials. The authors found that the thermal conductivity of 〈1‐10〉‐ and 〈110〉‐oriented GaN nanowires is substantially lower than that of the 〈001〉‐oriented ones.^[^ ^212^ ^]^

Recent findings suggested that the thermal properties of the thin‐film WBG materials have been successfully engineered using several innovative approaches, such as strain^[^ ^213^, ^214^, ^215^ ^]^ or buffer layer engineering.^[^ ^216^, ^217^ ^]^ For example, Tang et al. theoretically demonstrated that phonon properties of AlN/GaN/AlN nanofilms (e.g., group velocity) can be tailored by adjusting the value and direction of prestress to regulate the thermal performance of nanostructures.^[^ ^213^ ^]^ Yalamarthy et al. demonstrated the possibility of modulating electrical and thermal transport in the 2D electron gas at the AlGaN/GaN interface via buffer layer engineering. In particular, the variation of the GaN buffer layer can separately manipulate electrical transport and thermal conductance. The data show that the electrical conductivity in the thin GaN sample is identical to the bulk GaN sample (Figure 8e). By contrast, room temperature thermal conductivity dropped from ≈115 W m^−1^ K^−1^ for the bulk GaN sample to ≈45 W m^−1^ K^−1^ for the thin GaN sample due to the phonon boundary scattering. According to the authors, these differences between the electrical and thermal conductivity of the bulk and film GaN samples are achieved because electrons are at the heterostructured interface, while phonons are within the material system.^[^ ^216^ ^]^ While the coupling effects in electrical and thermal properties of WBG nanowire have been rarely reported, the interesting phenomena observed in the nanothin films could trigger new research direction in nanoscaled SiC, GaN, and diamond semiconductors.

## 6 Applications for Environmental Monitoring

### 6.1 Ultraviolet Sensors

UV sensors are among the most important devices which are widely used environmental monitoring.^[^ ^218^ ^]^ Photovoltaic UV sensors are generally classified into several types depending on their working principles, such as metal–semiconductor–metal, Schottky barrier, p–n junction, and p–i–n junction configurations.^[^ ^219^, ^220^ ^]^ UV sensors are characterized by a variety of important parameters, including cut‐off wavelength, photocurrent, dark current, time response, and quantum efficiency.^[^ ^218^ ^]^ The interesting optoelectrical properties of WBG semiconductor nanowires of SiC and group III‐nitrides have made them ideal building blocks for the development of high‐performance UV sensors bearing a superior level of sensitivity, stability, responsivity, response speed, and quantum efficiency.^[^ ^221^, ^222^, ^223^, ^224^, ^225^, ^226^ ^]^ For example, Wang et al. fabricated a Schottky contact device for visible‐blind UV detector using nonpolar *a‐*axial GaN nanowires. The UV detector displays a superior performance through a combination of its high sensitivity (up to 10^4^ A W^−1^) and external quantum efficiency (up to 10^5^), as well as ultrafast response speed (<26 ms).^[^ ^227^ ^]^

**Figure 9:**
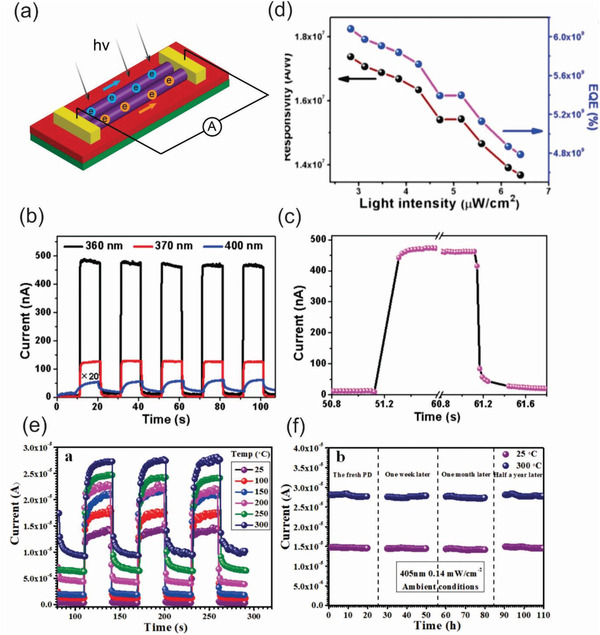
a) Schematic diagram of a bicrystalline GaN nanowire UV‐A photodetector. b,c) Reproducible on/off switching curves of the bicrystalline GaN nanowire UV‐A photodetector under different irradiation wavelengths from 360 to 400 nm at a 5 V bias voltage and the enlarged portion of 360 nm curve in (b). d) Light intensity‐dependent responsivity and external quantum efficiency at an excitation wavelength of 3600 nm under a bias of 5 V. (a)–(d) reproduced with permission.^[^ ^229^ ^]^ Copyright 2017, American Chemical Society. e) Spectral photoresponses of the single SiC nanobelt detector with 0.31 mol% B‐dopants as function of temperatures ranged from 25 to 300 °C measured at a bias of 5.0 V under the illumination of 405 nm light. f) The long‐term stability of as‐constructed detector up to 180 days under room temperature and 300 °C. (e) and (f) reproduced with permission.^[^ ^230^ ^]^ Copyright 2019, Wiley‐VCH.

Several approaches have been employed to enhance the overall performance of nanowire‐based UV sensors, such as surface modifications, doping, heterostructures, and bias voltage.^[^ ^228^, ^229^, ^230^, ^231^, ^232^, ^233^ ^]^ For example, Zhang et al. developed GaN nanowire photodetectors by the decoration of platinum (Pt) nanoparticles on the GaN nanowire surface, which exhibited a giant UV photoresponse. The peak responsivity and external quantum efficiency of the photodetector were increased from 773 to 6.39 × 10^4^ A W^−1^ and from 2.71 × 10^5^% to 2.24 × 10^7^%, respectively.^[^ ^228^ ^]^ The authors further improved the sensors performance using individual bicrystalline GaN nanowires (**Figure** **9**a). When under irradiation wavelength of 360, 370, and 400 nm, the GaN nanowires‐based UV detector showed an ultrasensitive response to 360 nm and the photocurrent increased significantly to a stable value and then sharply returned to its initial value as the source light is turned off as shown in Figure 9b,c. These results point out that the UV detector showed not only a significant *I*_on_/*I*_off_ ratio but also exhibited excellent repeatability. Moreover, the responsivity (*R_*λ*_*) and external quantum efficiency (EQE) were also derived from the experimental data (Figure 9d) based on equations: *R_*λ*_* = Δ*I*/*P* × *S* and EQE = *hc* × *R_*λ*_*/*eλ*, where Δ*I* is the difference between the photocurrent and the dark current, *P* is the light power, *S* is the irradiated area on the nanowire, *h* is the Plank constant, *c* is the velocity of the light, *e* is the electron charge, and *λ* is the incident light wavelength. The calculated results show that the responsivity and external quantum efficiency fall in the ranges of (1.37–1.74) × 10^7^ A W^−1^ and (4.79–6.08) × 10^9^%, respectively under 360 nm irradiation and 5 V bias voltage.^[^ ^229^ ^]^ Another way to improve the performance of UV sensors is via engineering doping elements. Very recently, Yang et al. fabricated high‐performance photodetectors based on a single B‐doped 3C‐SiC nanobelt.^[^ ^230^ ^]^ The detector showed a high responsivity and external quantum efficiency of 6.37 × 10^5^ A W^−1^ and 2.0 × 10^8^ under 405 nm light at 5 V bias voltage. To prove the operational stability in high‐temperature environment, the temperature‐dependent time responses of single B‐doped SiC nanobelt UV detector was measured under 405 nm light with a power density of 0.14 mW cm^−2^ at a bias of 5.0 V as shown in Figure 9e. As a result, the detector exhibited an excellent thermal stability and sensitivity, offering an interesting possibility for the detector to work under harsh environments. The long‐term stability of the device at both room and elevated temperatures for a period of ≈180 days was demonstrated with no observable degradation (Figure 9f).

For the commercial use of photodetectors, it is important to compare the performance of a series of devices fabricated from different WBG semiconductor nanowires. From material point of view, the performance of these devices is closely linked to physical characteristics of the chosen WBG semiconductor materials. For instance, the indirect bandgap of SiC‐based photodetectors causes a longer absorption length and longer minority carrier lifetime, while the direct bandgap of GaN‐based photodetectors leads to a short absorption length and a short lifetime. Most of the photocurrent of GaN‐based photodetectors is based on the generation of carriers in the depletion region. This results in a sharp absorption edge, enabling the maximization of the range of absorbed wavelengths, which can be considered as the main advantage of the GaN‐based photodetectors over other WBG counterparts.^[^ ^299^ ^]^ On the other hand, along with excellent electrical characteristics compared to other WBG semiconductors, SiC exhibits additional remarkable properties, such as radiation hardness, chemical inert, and mechanical stability, making SiC photodetectors suitable for extreme environments. In term of multiple layer architectures, III‐nitride materials offer an unprecedented platform to combine dissimilar materials in the same family or even with different elements, such as carbon‐based materials (e.g., graphene) to form various types of heterostructures. These new multilayered stacking architectures combined with advanced fabrication and growth techniques (e.g., molecular beam epitaxy and chemical vapor deposition), can significantly enhance the output of the III‐nitride based UV sensors.^[^ ^300^, ^301^, ^302^, ^303^ ^]^ Examples of this approach include the work of Martens et al., which developed an Al_0.25_Ga_0.75_N/GaN‐based UV photodetectors. The high conductivity of the 2D gas at the Al_0.25_Ga_0.75_N/GaN interfaces offers a high photocurrent in the milliampere‐range and high gain optical switch.^[^ ^300^ ^]^ Another study reported by Babichev et al. showed that the use of graphene as a contact for GaN nanowires can boost the UV responsivity up to ≈25 A W^−1^ at 357 nm at room temperature.^[^ ^303^ ^]^ In the case of diamond, although it is well‐known as the hardest, the most thermally conductive, and the highest in breakdown voltage material among WBG semiconductors, the implementation of diamond nanowires in UV photodetectors is extremely rare.^[^ ^311^ ^]^ This is because the synthesis of diamond nanowires in a reproducible way is still a challenging task due to the lack of a detailed understanding of the growth mechanism.^[^ ^312^ ^]^ Recently, several approaches based on chemical vapor deposition and reactive‐ion etching techniques have been employed to synthesis diamond nanowires, thus offering a promising pathway toward diamond‐based UV sensors.^[^ ^313^ ^]^ **Table** **2** summarizes several key parameters characterizing the operation performances of various photodetectors using WBG semiconductor nanowires and their heterostructures.

**Table 2:** Performance of various photodetectors using WBG semiconductor nanowires and their heterostructures

WBG nanowires (NWs)	Light [nm]	Photocurrent [A]	Dark current [A]	Responsivity [A W^−1^]	EQE/gain [%]	Response time [s]	References
3C‐SiC NWs	–	4.3 × 10^−6^	4.3 × 10^−8^	–	–	–	^[^ ^231^ ^]^
2H‐SiC NWs	375	1.8 × 10^−6^ – 2.7 × 10^−6^	–	–	–	<0.003	^[^ ^307^ ^]^
AlN NWs	193	2.4 × 10^−8^	1 × 10^−14^	0.39	254	<0.1	^[^ ^308^ ^]^
AlN NWs	325	–	–	2.7 × 10^6^	–	0.001	^[^ ^309^ ^]^
GaN NWs	320–400	–	–	1.74 × 10^7^	6.08 × 10^9^	0.144	^[^ ^229^ ^]^
GaN NWs	325	10^−8^	–	2.2 × 10^4^	3.2 × 10^7^	<0.026	^[^ ^227^ ^]^
GaN/AlN NWs	300	–	5.2 × 10^−14^	2 × 10^3^	–	–	^[^ ^232^ ^]^
GaN NWs/Pt	380	–	–	6.39 × 10^4^	2.24 × 10^7^	1.1	^[^ ^228^ ^]^
GaN NWs/graphene	357	1.28 × 10^−4^	–	25	–	–	^[^ ^303^ ^]^
AlN/GaN/AlN NWs	325	10^−9^–10^−12^	–	200–700	–	–	^[^ ^310^ ^]^
GaN/AlN NWs	280–330	0.5 × 10^−9^–3.4 × 10^−9^	–	3.1 × 10^6^–12 × 10^6^	–	–	^[^ ^233^ ^]^
Diamond NWs	300	3 × 10^−6^	7 × 10^−8^	338	–	0.02	^[^ ^311^ ^]^

### 6.2 Gas and Humidity Sensors

**Figure 10:**
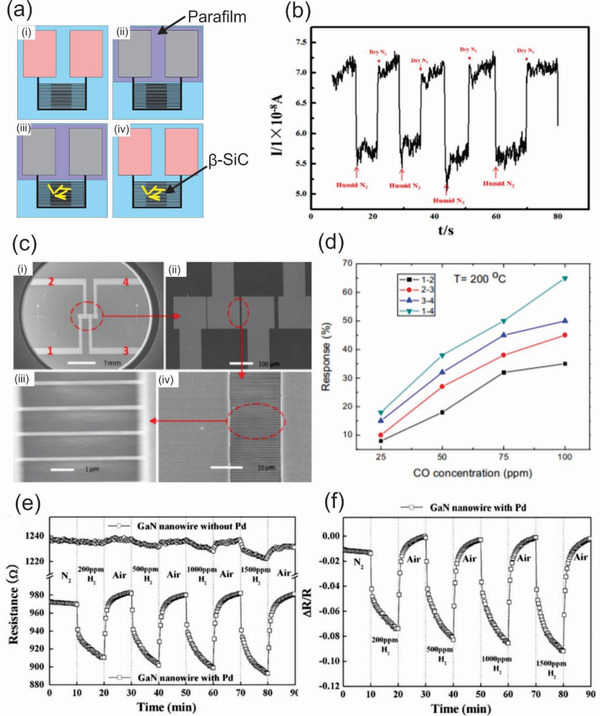
a) Schematic diagram of “titration‐coating” method: (i) original pad; (ii) pad with parafilm; (iii) covered with nanowires; (iv) removed parafilm. b) Response of electrode to humidity at room temperature. Reproduced with permission.^[^ ^234^ ^]^ Copyright 2019, the Amercian Ceramic Society. c) SEM images of the device: (i) the sample with four sputtered Au electrodes; (ii) the zoom‐in SEM image of Au electrodes; (iii) the high‐resolution image of the boron‐doped diamond nanowires between internal electrodes 2 and 3; (iv) the zoom‐in SEM image of (iii). d) Comparison of the responses of the three groups of diamond nanowires to CO as function of CO concentration. Reproduced with permission.^[^ ^53^ ^]^ Copyright 2017, Elsevier. e) Measured resistance at an applied bias of 0.5 V as a function of time from Pd‐coated and uncoated GaN nanowires devices exposed to a series of H_2_ concentrations for 10 min at room temperature. f) Relative response of Pd‐coated GaN nanowire devices as a function of time at different hydrogen concentrations. Reproduced with permission.^[^ ^243^ ^]^ Copyright 2009, Springer Nature.

Detecting gases and humidity are of interest for mining and energy industries. Gas sensors provide information regarding the quality of air as well as indicating the possibility of environmental pollution and catastrophic failures. In this context, WBG semiconductor nanowires of SiC, group III‐nitrides, and diamond are excellent candidates for fabricating gas and humidity sensors because their high surface‐to‐volume ratio makes the conductance of such nanowires highly sensitive to the changes in the surface state upon the adsorption of molecules. Several studies have been reported on the fabrication of various gas and humidity sensors employing different WBG semiconductor nanowires.^[^ ^234^, ^235^, ^236^, ^237^ ^]^ Shen et al. developed a humidity sensor based on the assembly of *β*‐SiC nanowires film by an interesting approach, namely “Titration‐coating” method (**Figure** **10**a). In this method, electrode pads were covered with a sealing film and subsequently an appropriate solution of *β*‐SiC nanowires was aspirated and spread onto electrode lines drying naturally. Finally, sealing film was removed after the nanowires had been allocated in desired areas. The humidity sensor performance was apparently demonstrated by the typical humidity response/recovery property of *β*‐SiC nanowires examined in humid/dry N_2_ atmosphere (Figure 10b). The current decreased and increased rapidly when turning on humid N_2_ and pure N_2_. The curves hardly changed after several cycling tests, showing an excellent stability of the sensor.^[^ ^234^ ^]^

Numerous theoretical frameworks have been developed to understand the operating principle of WBG nanowire electrical conductance.^[^ ^31^, ^34^ ^]^ At a given temperature, the conductance of semiconductor nanowires can be calculated as *G* = *n*_0_ × *e* × *μ* × *d* ^2^/*L* where *n*_0_ is the original carrier concentration, *e* is the electronic charge, *μ* is the mobility of the electrons, while *d* and *L* are radius and length of the nanowire channel, respectively. The adsorption of molecular gases results in the changes of the conductance induced by the change in carrier concentration Δ*n*_s_. This is due to a variation of surface carrier given by Δ*G* = Δ*n*_s_ × *e* × *μ × d* ^2^/*L*. Therefore, the sensitivity of sensors can be defined as Δ*G*/*G* = Δ*n*_s_/*n*_0_.^[^ ^31^, ^34^, ^238^, ^239^ ^]^ Based on this relatively simple model, the sensitivity of gas and humidity sensors can be tailored by properly decorating the surface of nanowires with appropriate amounts of noble nanoparticles as catalysts to accelerate the surface reactions or modifying the intrinsic properties of nanowires using surface treatments, or introducing impurities in another pathway to boost up the device performance.^[^ ^240^, ^241^, ^242^, ^243^ ^]^ For instance, Peng et al. developed high‐performance gas sensor arrays for detecting carbon monoxide based on boron‐doped ultra‐nanocrystalline diamond nanowires.^[^ ^53^ ^]^ Figure 10c illustrates a prototype of the gas sensor arrays, consisting of four sputtered Au electrodes and the boron‐doped diamond nanowires. Three different groups of boron‐doped diamond nanowires were employed for the fabrication of the sensors. The experimental results revealed that the responses of the three sensors increased with the CO concentrations, as shown in Figure 10d. In another example, Johnson et al. demonstrated that the coating of GaN nanowires with thin layer of Pd is an effective way to obtain a high‐performance gas sensor.^[^ ^243^ ^]^ Hydrogen sensing experiments using the Pd‐coated‐GaN nanowire‐based sensors displayed a high sensitivity response and an excellent recovery at room temperature compared to that of the uncoated GaN nanowire‐based gas sensors (Figure 10e,f).

It should be noted that, nanowires of SiC and group III‐nitrides are more widely employed in producing high‐performance gas and humidity sensing devices compared to diamond nanowires. The reason for this is similar to the case of UV photodetectors as previously mentioned. Recently, a promising sensing technology using AlGaN/GaN high electron mobility transistors is attracting a great deal of attention, particularly for commercial applications, due to their high mobility and saturation velocity in the 2DEG layer. In such transistors, the conducting 2DEG channel is extremely close to the device surface and therefore highly sensitive to adsorption of analytes. This feature enables these gas sensing devices for detecting a broad variety of gases, which might be not accessible for sensors fabricated using the conventional architectures.^[^ ^313^ ^]^

### 6.3 Strain Sensors

**Figure 11:**
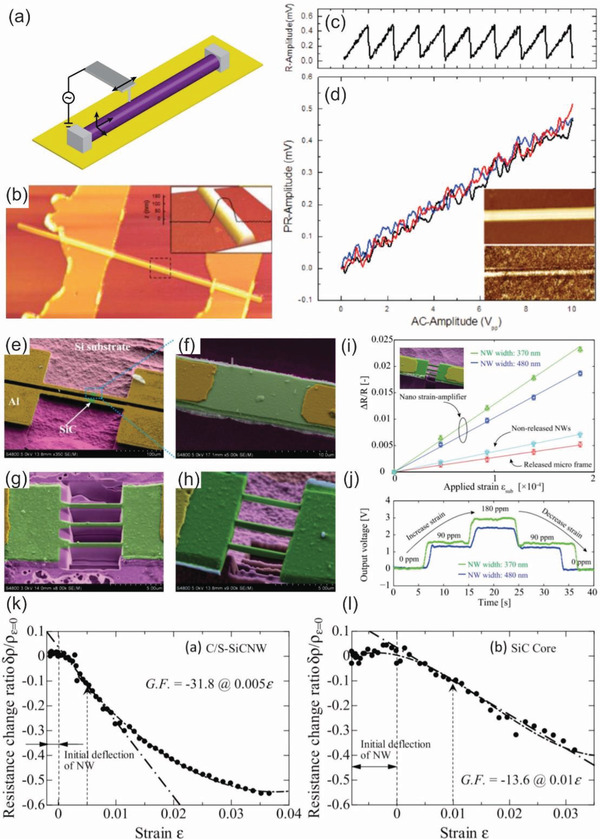
a) Schematic showing one of the two configurations for probing the 3D piezoelectric tensor of a single *c*‐axis GaN nanowires. b) A taping mode AFM image of the nanowire and electric contacts. The inset in (b) shows a magnification image of the area boxed with dashed lines showing the top surface of the nanowire. c) Nine response curves obtained in the axial direction at a single point on the nanowire. d) Piezoresponse amplitude acquired at three different points on the same nanowire, showing repeatability of response along its axial direction. (a)–(d) reproduced with permission.^[^ ^250^ ^]^ Copyright 2012, Amercian Chemical Society. e–h) SEM images of the SiC strain sensors: (e) Released SiC microbridge used for the fabrication of the nanostrain‐amplifier, (f) SEM image of a micro‐SiC resistor where the SiC nanowires array were formed using FIB, (g) SEM of nonreleased SiC nanowires, and (h) SEM of locally fabricated SiC nanowires released from the Si substrate. i) A comparison between the relative resistance change in the nanostrainamplifiers, nonreleased nanowires, and released microframes and j) the repeatability of the SiC nanowires strain sensors using the proposed structure. (e)–(j) reproduced with permission.^[^ ^253^ ^]^ Copyright 2016, AIP Publishing. k,l) Variations of the resistance charge ratio with an increase of the tensile strain for the core–shell SiC nanowires and for the SiC core without the SiOx shell, respectively. (k) and (l) reproduced with permission.^[^ ^254^ ^]^ Copyright 2019, IOP Publishing.

Strain sensors are among the most important classes of piezoresistive sensors, which are based on the change in electrical resistivity of a material subjected to applied mechanical stress.^[^ ^244^, ^245^ ^]^ For mechanical sensing in extreme conditions, WBG semiconductor nanowires of SiC, group III‐nitrides, and diamond are the material of choice due to their outstanding electrical properties, chemical inertness, mechanical durability and especially, a tunable giant piezoresistive effect.^[^ ^246^, ^247^, ^248^, ^249^, ^250^ ^]^ For instance, Gao et al. reported the piezoresistance behaviors of single p‐type 6H‐SiC nanowires under different compressive stresses using a conductive AFM as the test bed.^[^ ^246^ ^]^ The authors observed the decrease of the nanowire resistance with increasing compressive strain. The transverse piezoresistance coefficient *π* ^[010]^ of the nanowire was calculated to be in the range of 51.2 to 159.5 × 10^−11^ Pa^−1^ under the applied loading forces from 43.8 to 140.2 nN. Jolandan et al. presented a method based on scanning force microscopy to obtain the 3D piezoelectric matrix of individual GaN nanowires.^[^ ^250^ ^]^ **Figure** **11**a shows one of the two configurations for the experimental setup used in this work, in which the GaN nanowires were mounted onto a Si substrate coated with a conductive Au layer. The electric field is applied between the tip of the conductive AFM probe and the grounded substrate. Figure 11b shows a tapping mode AFM topography image of the GaN nanowire and electric contacts. The measurements of the piezoresponse for a nanowire were conducted by applying the AC voltage between two electrodes and the torsional twist of the AFM cantilever monitors the induced piezoresponse in the axial direction of the nanowire. Figure 11c,d shows piezoresponse curves obtained at a single point and three different points on the same GaN nanowire, respectively, which unambiguously indicate repeatability of response along the axial direction. Based on their experimental results, the authors found that GaN nanowires exhibit strong piezoelectricity in three dimensions, with up to six times larger than the effect in bulk.

Recently, several methods based on both top‐down and bottom‐up approaches have been employed to enhance the sensitivity of piezoresistive strain sensors for wider applications.^[^ ^251^, ^252^, ^253^, ^254^ ^]^ In this regard, our group reported a revolutionary mechanical approach to enhance the sensitivity of piezoresistive strain sensors using a 3C‐SiC nanowire‐based strain amplifying structure (namely, nanostrain‐amplifier).^[^ ^253^ ^]^ The nanostrain‐amplifier was inspired from the dogbone structure. It enables to magnify the strain in the desired areas. Figure 11e–h shows the SEM images of the fabricated samples including the conventional released structure, nonreleased nanowires and the nanostrain‐amplifier. Figure 11i shows the relative resistance change (Δ*R*/*R*) of the micro‐ and nano‐SiC resistors as a function of the applied strain *ε*_sub_. With the similar applied strain to the Si substrate, the resistance changes of the SiC nanowires using the nanostrain‐amplifier was much larger than that of the SiC microresistor and the conventional nonreleased SiC nanowires. More interestingly, the authors found that the reduced width of the SiC nanowires increases the sensitivity. Based on their experimental data, the effective gauge factor of the 380 and 470 nm SiC nanowires was determined as 150 and 124, respectively, which means that the sensitivity of the nanostrain‐amplifier was enhanced by 5.3 times and 4.6 times in comparison to that of the bulk SiC, respectively. These findings unambiguously demonstrated that the nanostrain‐amplifier is an ideal platform for the fabrication of ultrasensitive strain sensors. Very recently, Nakata et al. reported another interesting method for improving the sensitivity of sensing devices operating in harsh environments.^[^ ^254^ ^]^ This method is based on the bottom‐up approach in which the core–shell SiC nanowires were synthesized by a VLS technique. Figure 11j,k shows the variations of the resistance change ratio depending on an increase of the tensile strain for the core–shell SiC nanowires and SiC core, respectively. The experimental results show a gauge factor of −30.7 at 0.008 *ε* for the core–shell SiC nanowires approximately two times larger than that of −15.8 at 0.01 *ε* for the SiC core. The authors hypothesized that an increase of the surface state density at the SiO*_x_*/SiC interface due to the positive fixed oxide charge of the SiO*_x_* shell could be the reason behind the enhancement in the piezoresistance of the core–shell nanowires.

Among different classes of WBG materials, SiC is the preferable choice for strain sensing owing to its tolerance to elevated temperatures, UV radiation, and chemical corrosion. Interestingly, the operating temperature of SiC was reported as high as 800 °C together with the increasing gauge factors when temperatures increased from 500 to 800 °C while III‐nitride based sensors have also been developed, although their working temperatures might be not as high as SiC‐based sensors.^[^ ^314^, ^315^ ^]^ On the other hand, diamond is also an interesting material for high‐temperature strain sensors due to its piezoresistive nature as well as other unique mechanical, thermal, and electrical properties. However, the piezoresistive properties of diamond strongly rely on a number of factors, such as the quality of crystalline structures, defects, and doping level. Consequently, diamond nanowires‐based strain sensors are still in a nascent stage of technical development and thus, more investigations are urgently needed in the coming years.

### 6.4 Pressure Sensors

**Figure 12:**
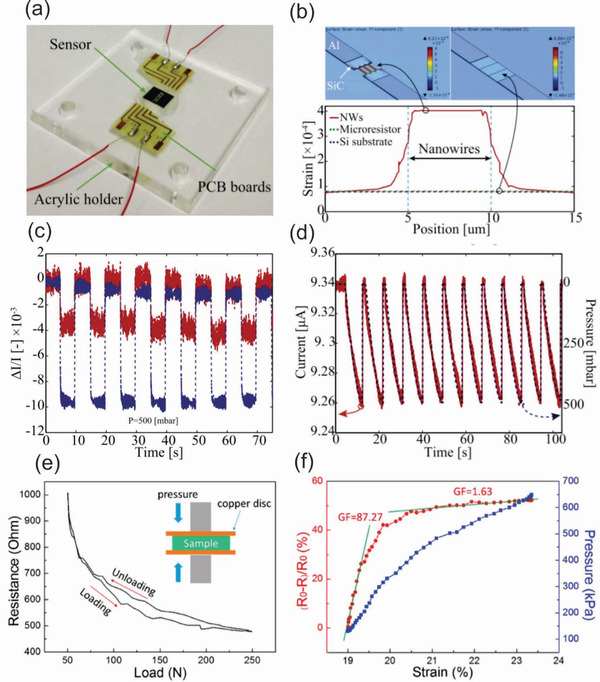
a) Photograph of SiC nanowire‐based pressure sensor mounted on an acrylic holder. b) Simulation of strain induced into the nanowires and microscaled frames. c) A threefold increase in the sensitivity of nanowire sensors (blue) in comparison to SiC microresistors (red). d) The response of the nanowire pressure sensors under ram‐up pressures from 0 to 500 mbar. (a)–(d) reproduced with permission.^[^ ^261^ ^]^ Copyright 2018, Elsevier. e) Electrical resistance as a function of applied compressive stress. Inset in (e) shows the schematic setup for the pressure‐dependent electrical response measurement. f) Variation of electrical resistance change and applied pressure with compressing cycles. Gauge factors were derived from linear fitting. (e) and (f) reproduced with permission.^[^ ^262^ ^]^ Copyright 2019, American Chemical Society.

Similar to the case of strain sensors, piezoelectric and piezoresistive effects are also widely applied in pressure sensors for deep sea and space exploration as well as monitoring industrial processing (e.g., pressure in combustion energies, or pressure in chemical reaction chambers).^[^ ^255^ ^]^ So far, a considerable number of studies has been reported on the development of pressure sensors employing different types of materials, possessing either an excellent piezoelectric property or a coupling of piezoelectric and semiconducting properties.^[^ ^256^, ^257^, ^258^, ^259^, ^260^ ^]^ However, the fabrication of high‐performance pressure sensors based on WBG semiconductor nanowires, especially for SiC, group III‐nitrides, and diamond, are very rare.^[^ ^261^, ^262^, ^263^ ^]^ For instance, Phan et al. reported an effective approach to develop highly sensitive pressure sensors employing 3C‐SiC nanowires fabricated at the center of a dogbone‐like structure.^[^ ^261^ ^]^ **Figure** **12**a shows a photograph of SiC nanowire‐based pressure sensors mounted on an acrylic holder. The authors performed a finite element analysis to estimate the strain induced into the SiC nanowires and suspended microbridge (Figure 12b). The team found that the strain induced into the as‐fabricated nanowires was almost five times larger than that of the microresistors. The experimental results of the response of the sensors subjected to cyclic square‐pressure of 500 mbar are shown in Figure 12c. Evidently, the response of the nanowire‐based pressure sensors was approximately three times larger than that of the pressure sensors using microsized SiC, which was in a good agreement with their simulation results. Figure 12d shows the output of the nanowire pressure sensors against ramped‐up pressure input, indicating fast time response and good repeatability with a variation of less than 3% between cycles. Very recently, Chen et al., developed lightweight SiC nanowire sponges via the simple sugar‐blowing‐assisted carbothermal reduction.^[^ ^262^ ^]^ Their experimental results showed an excellent pressure‐dependent electrical response. The electrical resistance decreased with the rise of the applied load but completely recovered upon unloading (Figure 12e). Meanwhile, the sponge exhibited abnormally high sensitivity: the strain gauge factor went up to 87.27 at the beginning of the test with strain change of no more than 1% and then dropped to 1.63 in the high‐strain range of 20.5% to 23.35% (Figure 12f).

For comparison, both SiC and group III‐nitrides are good materials for pressure sensors using either the piezoresistive or piezoelectric effect. By contrast, diamond is a nonpiezoelectric material due to their inherent symmetry and consequently, it is not suitable for the fabrication of pressure sensors based on the piezoelectric properties.^[^ ^316^ ^]^ Notably, piezoresistive‐based sensors exhibit higher sensitivity and better low‐frequency response compared to piezoelectric‐based sensors. Thus, the choice of sensing materials is strongly dependent on the targeted applications of the pressure sensors. **Table** **3** summarizes important parameters of piezoelectric materials of SiC and group III‐nitrides.^[^ ^317^ ^]^ In general, group III‐nitrides, especially GaN and AlN, are the most common materials used in producing piezoelectric‐based pressure sensors among various WBG semiconductor materials. Particularly, AlGaN/GaN heterostructures offer a huge advantage over other WBG materials for pressure sensors operating at high‐frequency and high‐power operations due to the ability to achieve 2D electron gases without intentional doping.^[^ ^316^, ^318^ ^]^

**Table 3:** A comparison of the piezoelectric effect in common WBG materials^[^ ^317^ ^]^

Materials	SiC	GaN	AlN	Sc‐doped AlN
Elastic modulus, *c*_33_ [GPa]	605	398	390	390
Acoustic velocity [m s^−1^]	13 100	8044	11 000	8509
Piezoelectric constant, *e*_33_ [C m^−2^]	+0.2	+0.65	+1.55	+3.9
Electromechanical coupling coefficient, *k* ^2^ [%]	0.08	2	5.6	15.5

### 6.5 Nanogenerators for Energy Harvesting

**Figure 13:**
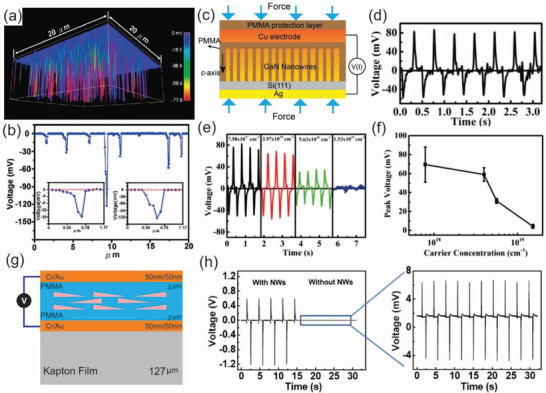
a) 3D plot of the output voltage recorded at an external load when the AFM tip scanned and deflected the nanowire arrays. Reproduced with permission.^[^ ^277^ ^]^ Copyright 2010, Amercian Chemical Society. b) A typical line scan profile of the output voltage. Insets in (b) show the detailed line profile of individual output peaks. Reproduced with permission.^[^ ^277^ ^]^ Copyright 2010, Amercian Chemical Society. c,d) Schematic illustration of the structure of GaN nanowires‐based nanogenerators and the output voltage of the device, respectively. Reproduced with permission.^[^ ^285^ ^]^ Copyright 2014, Wiley‐VCH. e,f) Output voltage and extracted average output voltage of four nanogenerators with n‐GaN arrays with different carrier concentrations. Reproduced with permission.^[^ ^285^ ^]^ Copyright 2014, Wiley‐VCH. g) Schematic illustration of GaN nanowires‐based flexible nanogenerators and h) a comparison of output voltages between the flexible nanogenerators with and without GaN nanowires. Reproduced with permission.^[^ ^291^ ^]^ Copyright 2011, IOP Publishing.

To date, most of the sensing devices for environmental monitoring still rely on conventional power sources, such as rechargeable batteries or supercapacitors. However, replacing of the battery for individual components integrated into a sensor network is time‐consuming and expensive and thus, limiting the sustainable operation of sensing devices, especially for large‐scale remote applications. Moreover, materials used to produce batteries may lead to negative impacts on heath and environments. Consequently, tremendous efforts in recent years have been committed to designing and constructing viable wireless sensing devices with a self‐powered ability for generating the next‐generation sensor networks.^[^ ^264^, ^265^, ^266^, ^267^ ^]^ In this regard, the piezoelectric nanogenerators converting mechanical energy into electric energy are currently viewed as the most effective energy harvesters for powering wireless sensing devices due to their feasibility to integrate into complex sensor networks and capability of direct conversion.^[^ ^268^, ^269^, ^270^, ^271^, ^272^, ^273^ ^]^ Among numerous piezoelectric materials, nanowires bearing wurtzite crystal structure of group III‐nitrides such as GaN, AlN, and InN are the most attractive candidates for the fabrication of such piezoelectric nanogenerators.^[^ ^274^, ^275^, ^276^, ^277^, ^278^ ^]^ The Wang group is among the pioneers in this field and has successfully demonstrated that epitaxial GaN nanowires on GaN/sapphire substrates can function as building blocks for the construction of high‐output nanogenerators.^[^ ^277^ ^]^ Wang and colleagues employed AFM in contact mode for the piezoelectric measurements and found that the average of piezoelectric output voltage was about −20 mV, while 5% to 10% of the GaN nanowires exhibited piezoelectric output voltages exceeding −100 mV (**Figure** **13**a,b).

Controlling and improving the output voltage of piezoelectric nanogenerators is one of the necessary prerequisites for the successful application of self‐powered sensing devices in diverse practical purposes. With this in mind, researchers have never ceased to seek new strategies for the enhancement of piezoelectric properties for the development of high‐output nanogenerators. In this regard, numerous methods have been developed to enhance the output voltage of piezoelectric nanogenerators based on various types of materials, such as ZnO nanowires.^[^ ^279^, ^280^, ^281^, ^282^ ^]^ An investigation into SiC and group III‐nitride nanowire based nanogenerators has been incorporated into this topic as these materials offer more stable performance than ZnO under hostile conditions. Xu et al. compared the electrical potential of n‐doped GaN–AlN–GaN nanowires and intrinsic GaN nanowires using AFM.^[^ ^283^ ^]^ The authors found that the measured electric potential of n‐doped Gan–AlN–GaN was 200 mV, while that of intrinsic GaN nanowires was 150 mV. Their findings point out that the combination of different group III‐nitrides nanowires could be a promising protocol to enhance the piezoelectric output voltage for the future nanogenerators. In another work, Liu et al. fabricated piezoelectric nanogenerators based on vertically aligned InN nanowires.^[^ ^284^ ^]^ The authors compared the output performance between p‐type and intrinsic InN nanowires and found that the p‐type nanowires show 160% more output current and 70% more output power than the intrinsic one. This finding demonstrated that doping plays an important role in the piezoelectricity of nanogenerators. This hypothesis was later experimentally demonstrated in GaN nanowire with tunable free carrier concentration.^[^ ^285^ ^]^ In that work, a series of Si‐doped GaN nanowire arrays were employed to investigate the carrier screening effect on the output voltage of the nanogenerators. Figure 13c shows the structure of this device where GaN nanowire arrays with a doping concentration of 7.58 × 10^17^ cm^−3^ were assembled into vertically integrated nanogenerator. Under cyclic pressing and releasing at 2 Hz with a maximum force of about 5 kgf, the resulting output voltage was ≈80 mV (Figure 13d). By varying the carrier concentrations from 7.58 × 10^17^ to 1.53 × 10^19^, the authors found that the average peak output voltage of the nanogenerator dropped from ≈70 mV for the lightly doped device gradually down to ≈4 mV for the heaviest doped device (Figure 13e,f).

To promote wearable sensing devices for diverse applications, the integration of flexibility and stretchability into nanogenerators is of paramount importance.^[^ ^286^, ^287^, ^288^, ^289^, ^290^ ^]^ In this context, several flexible nanogenerators based on group III‐nitrides nanowires having high‐output performance have been successfully developed in recent years.^[^ ^291^, ^292^ ^]^ For instance, Lin et al. fabricated a nanogenerator by an assembly of GaN nanowires on a flexible substrate.^[^ ^291^ ^]^ Figure 12g shows the schematic illustration of the structure of the GaN nanowires‐based nanogenerator. The authors found that the flexible nanogenerators fabricated by rational assembly of GaN nanowires exhibited an output voltage up to 1.2 V. Importantly, the authors performed a control experiment comparing the electrical output of the nanogenerator with and without GaN nanowires. The experimental results show that the output voltage of the devices without GaN nanowires was much lower than that of the devices fabricated with GaN nanowires (Figure 13h) and thus, unambiguously proving the vital role of GaN nanowires in the development of high‐output nanogenerators. More recently, Johar et al. reported the fabrication of a flexible piezoelectric nanogenerators based on GaN nanowires transferred on a Si‐rubber substrate.^[^ ^292^ ^]^ Their experimental results show that the output voltage of the flexible nanogenerator was 15.4 V at actuation frequency of 8 Hz. Moreover, the flexible nanogenerator exhibited long‐term stability with a degradation rate of less than 18% of its initial output after 20 000 cycles.

## 7 Conclusion and Perspective

The last few decades have seen rapid growth in the field of WBG semiconductor nanowires, from material processing, fundamental characterization to practical device development. A wide range of bottom‐up and top‐down approaches has been intensively employed to synthesize SiC, group III‐nitride, and diamond nanowires as building blocks for diverse sensing devices. Each approach has its particular merits and inevitable disadvantages. Bottom‐up nanowires growth methods offer great flexibility to achieve sophisticated nanostructures with complex compositions which may not be accessible by top‐down methods. By contrast, top‐down processes allow obtaining highly ordered arrays of semiconductor nanowires with perfect uniformity and reproducibility for the wafer‐scale fabrication of sensing devices. From a practical standpoint, we expect that a well‐thought mix of both bottom‐up and top‐down approaches will be the future route to develop innovative nanowire‐based sensing devices bearing unprecedented performances which may not be feasible by either technique alone. Moreover, a more thorough understanding of the growth mechanism, further optimizing the growth parameters and continued improvements of the advanced nanomachining techniques should be constantly given a high priority to precisely control the crystal structure, dimensions, phase purity, interfaces, defects, compositions, and orientations of nanowires. This is prerequisite to establish a basic platform for the incremental developments of the future nanowire‐based sensing devices as well as their integration into standardized electronics.

It should be noted that intrinsic remarkable properties of semiconductor nanowires can be further enhanced for their integrations into high‐performance multifunctional sensing devices. In this context, several efficient protocols have been successfully developed to enhance the properties of various semiconductor nanowires, such as doping, surface modifications, combining different materials to create heterostructures, applying external strains. However, compared to the well‐known Si nanowires, the improvements in properties of WBG semiconductor nanowires focused in the framework of this review are still in an early stage of technical development and thus, more research toward these nanowires are urgently needed. Besides, it is vital to continuously seek new and cost‐effective pathways to further enhance the long‐term stability, selectivity, and sensitivity of nanowire‐based sensing devices. For instance, surface functionalization of the nanowires with molecular adsorbates could be a promising alternative pathway to conventional volume doping for modulating the conductivity of the nanowires for the development of the next generation sensing devices. The key merit of this approach is that there is an almost infinite variety of molecular adsorbates that can be synthesized at a reasonable cost, which offers a huge flexibility to achieve a new class of functionalized nanowires with programmable properties obtained by controlling the charge transfer at the nanowire/adsorbate interfaces. In this regard, an in‐depth understanding of the mechanisms of the surface effect on the nanowires upon the adsorption of molecular adsorbates is urgently needed for successful application of this approach.

For mechanical sensing applications, the piezoresistive and piezoelectric effects have been utilized as fundamental sensing principles. Compared to Si nanowires, these sensing phenomena in SiC, group III‐nitrides, and diamond nanowires have not been fully understood due to the lack of comprehensive theoretical and experimental studies. For example, unlike Si nanowires, very few studies on a giant piezoresistive effect in such classes of nanowires have been reported till date. Thus, more investigations to establish a reliable protocol to control and enhance the piezoresistive effects of these nanowires is highly recommended, particularly for applications in harsh environments where Si cannot be used. Moreover, the stability of the piezoresistive effect when the sensing devices are exposed to extreme conditions, such as high temperature, solar radiation, acidic rain, corrosion, oxidation should be taken into considerations to guarantee long‐term operation of nanowire‐based sensing devices. This is of topmost importance for the transition of such sensing devices from the laboratory to commercial and industrial products. Recently, the piezoelectric nanogenerators have been rapidly emerging as sustainable tools to power‐up distributed sensor networks for environmental applications. Despite remarkable progress has been made in this field of self‐powered microelectronic devices, the low output performance and lack of long‐term stability of nanowire‐based nanogenerators, especially under hostile conditions, remain significant challenges for their practical uses. Several methods have been proposed to overcome these significant challenges, but they are still in a nascent stage of technical developments. An insight into various intrinsic and extrinsic parameters determining the variations of the output voltage of nanogenerators should be placed at the center of the research efforts in the coming years.

With the continuous development of science and technology, sensing and microelectronic devices have become increasingly important for every aspect of our daily lives. Advanced nanomachining techniques offer a huge advantage to design and construct an array of sensing devices with complex architectures. For instance, the integration of flexibility and stretchability to nanowires for developing wearable and portable self‐powered sensing devices is expected to broaden their breadth of applications. Besides, the integration of different nanogenerators and sensing elements into a single sensor network could be an ideal choice in the era of IoTs. The recent advancements in smart city, IoTs, big data analytics, and machine learning can help to interconnect sensing components into a giant sensor network in which all sensing nodes are capable of collecting and sharing as well as self‐learning based on feedback signals. These advanced technologies are extremely beneficial to improve and optimize the efficiency of the next generation of NEMS with wide bandgap materials at their heart.

## Conflict of Interest

The authors declare no conflict of interest.
